# A Pilot Study Evaluating the Safety and Clinical Use of a Leg Exercise Apparatus in Patients With Stroke or Spinal Cord Injury: A Comparison With an Ergometer

**DOI:** 10.7759/cureus.91357

**Published:** 2025-08-31

**Authors:** Nobuhiro Hirasawa, Yukiyo Shimizu, Ryu Ishimoto, Hirotaka Mutsuzaki, Yasushi Hada

**Affiliations:** 1 Department of Rehabilitation Medicine, Ibaraki Prefectural University of Health Sciences Hospital, Ami, JPN; 2 Department of Rehabilitation Medicine, Institute of Medicine, University of Tsukuba, Tsukuba, JPN; 3 Center for Medical Science, Ibaraki Prefectural University of Health Sciences, Ami, JPN; 4 Department of Orthopaedic Surgery, Ibaraki Prefectural University of Health Sciences Hospital, Ami, JPN

**Keywords:** leg exercise, leg exercise apparatus, paralysis, spinal cord injury, stroke, venous thromboembolism

## Abstract

Background and objectives: The leg exercise apparatus (LEX), initially developed to prevent thrombosis, may also serve as a rehabilitation exercise device due to its ability to promote active movement. This pilot study aimed to evaluate the safety and clinical indications of LEX exercises in patients with stroke or spinal cord injury (SCI).

Materials and methods: Nine patients with stroke or SCI participated in this study. Participants were assessed for paralysis, muscle strength, sensation, range of motion (ROM), spasticity, vital signs, and modified Borg scale (mBS) scores for respiratory and lower extremity fatigue. Each participant performed LEX and ergometer exercises for five minutes, with the number of repetitions recorded for both. After each session, ROM, spasticity, vital signs, and mBS were re-evaluated. After completing all exercise sessions, participants completed a questionnaire regarding their experience with each device and reported any adverse events. The primary outcome was the occurrence of adverse events within one hour following LEX exercise.

Results: Two patients with SCI reported transient pain attributed to device contact or increased spasticity that resolved within an hour. During the LEX exercise, two participants required assistance, primarily to prevent the hip joint from excessive abduction. Three participants required assistance with the ergometer, including the prevention of excessive hip abduction and avoiding reverse pedaling. One participant required both types of assistance and discontinued the ergometer exercise midway. The mBS scores indicated lower fatigue levels with LEX compared to the fatigue levels with the ergometer, likely due to difficulties in controlling rotational movement with the latter. Therefore, LEX appeared to be more suitable than the ergometer for sustained exercise in patients with motor impairments such as stroke or SCI.

Conclusions: This study demonstrated that LEX is safe for patients with stroke or SCI, with only minor transient adverse events. With slight modifications to improve stability and usability, LEX may be a practical and effective option for lower limb rehabilitation in patients with motor impairments.

## Introduction

Patients with lower extremity paralysis, including those with stroke or spinal cord injury (SCI), are at increased risk of venous thromboembolism (VTE) due to prolonged immobility [1,2]. To prevent VTE, both pharmacological and mechanical thromboprophylaxis are often used [3,4]. Mechanical prophylactic devices, such as elastic compression stockings and intermittent pneumatic compression, are classified as passive devices because they function without active movement. As prolonged bed rest not only increases the risk of VTE but also leads to other adverse effects, such as muscle atrophy in the lower extremities due to disuse [5], passive thromboprophylactic devices alone are insufficient for preventing these complications.

To address this limitation, the University of Tsukuba in Tsukuba, Japan, developed the leg exercise apparatus (LEX), an active thromboprophylactic device. The LEX can be placed on a bed, secured to the foot end of the frame with hooks, and used while the patient is in a supine position. The device facilitates lower-extremity movements, including dorsiflexion, plantar flexion, ankle eversion and inversion, and multi-joint leg movements. Previous studies have demonstrated the usefulness of LEX in thromboprophylaxis, showing improvements in venous blood flow and safe use in some patient populations, and its active mechanism also suggests potential applications in rehabilitation [6-10]. Previously, muscle activity during LEX was investigated and compared with active leg movement without LEX and with an ergometer [11,12]. These studies showed that the LEX stimulates efficient activity of the triceps and other lower extremity muscles.

Patients with stroke or SCI are at a high risk of developing VTE. Additionally, their ability to engage in leg exercise is often limited due to motor disorders and an inability to walk. The LEX may serve not only as a VTE prophylactic device but also as an exercise tool for these patients. However, there are few reports on the use of LEX in actual patients. Existing studies have focused primarily on patients who have undergone total joint arthroplasty of the lower extremities or patients with spinal disease, mainly vertebral fractures [6,9]. Notably, no studies have investigated the use of LEX in patients with motor impairments, including partial or complete paralysis.

Patients with stroke or SCI must also be mindful of circulatory changes, including recurrent hemorrhage or orthostatic hypotension. Previously, circulatory dynamics during LEX exercises were investigated and compared with those of ergometers [12]. As a result, LEX was not clinically different from ergometer exercise in terms of circulatory dynamics. However, its safety in these patients has not yet been established.

Therefore, to address such gaps in evidence regarding the use of LEX in patients with stroke or SCI, we conducted a pilot study comparing LEX with ergometers in this population to evaluate its safety and identify the conditions under which it may be indicated.

## Materials and methods

Ethical considerations

The study was conducted in accordance with the principles of the Declaration of Helsinki. Prior to its commencement, approval was obtained from the Institutional Review Board (IRB) of the Ibaraki Prefectural University of Health Sciences, Ami, Japan (approval no. 1175), ensuring that all procedures adhered to rigorous ethical standards. The IRB review included a detailed assessment of the study's objectives, methodology, potential risks and benefits, participant privacy protections, and the informed consent process. Written informed consent was obtained from all participants before their inclusion in the study. Participants were informed about the study's nature, its potential risks and benefits, and their right to withdraw at any time without penalty. The informed consent process ensured that participants had ample opportunities to ask questions and receive comprehensive explanations before agreeing to participate.

Participants

The inclusion criteria were volunteers diagnosed with stroke or SCI who were admitted to the Ibaraki Prefectural University of Health Sciences Hospital for rehabilitation and were recruited through an in-hospital call; recruitment was non-random and non-consecutive. Several exclusion criteria were established to ensure the exercise could be performed properly and safely. These included patients who were deemed unable to perform appropriate exercises due to severe cognitive or higher brain dysfunction, as determined by clinical assessment; patients with serious complications such as cardiac, hepatic, or renal disease; individuals who were pregnant or suspected to be pregnant; patients who lacked the capacity to provide voluntary consent; and any other individuals deemed inappropriate by the physician in charge. No minimum threshold for lower-extremity muscle strength was set, as the study aimed to examine the feasibility of the exercise across different levels of motor impairment.

Intervention

**Figure 1:**
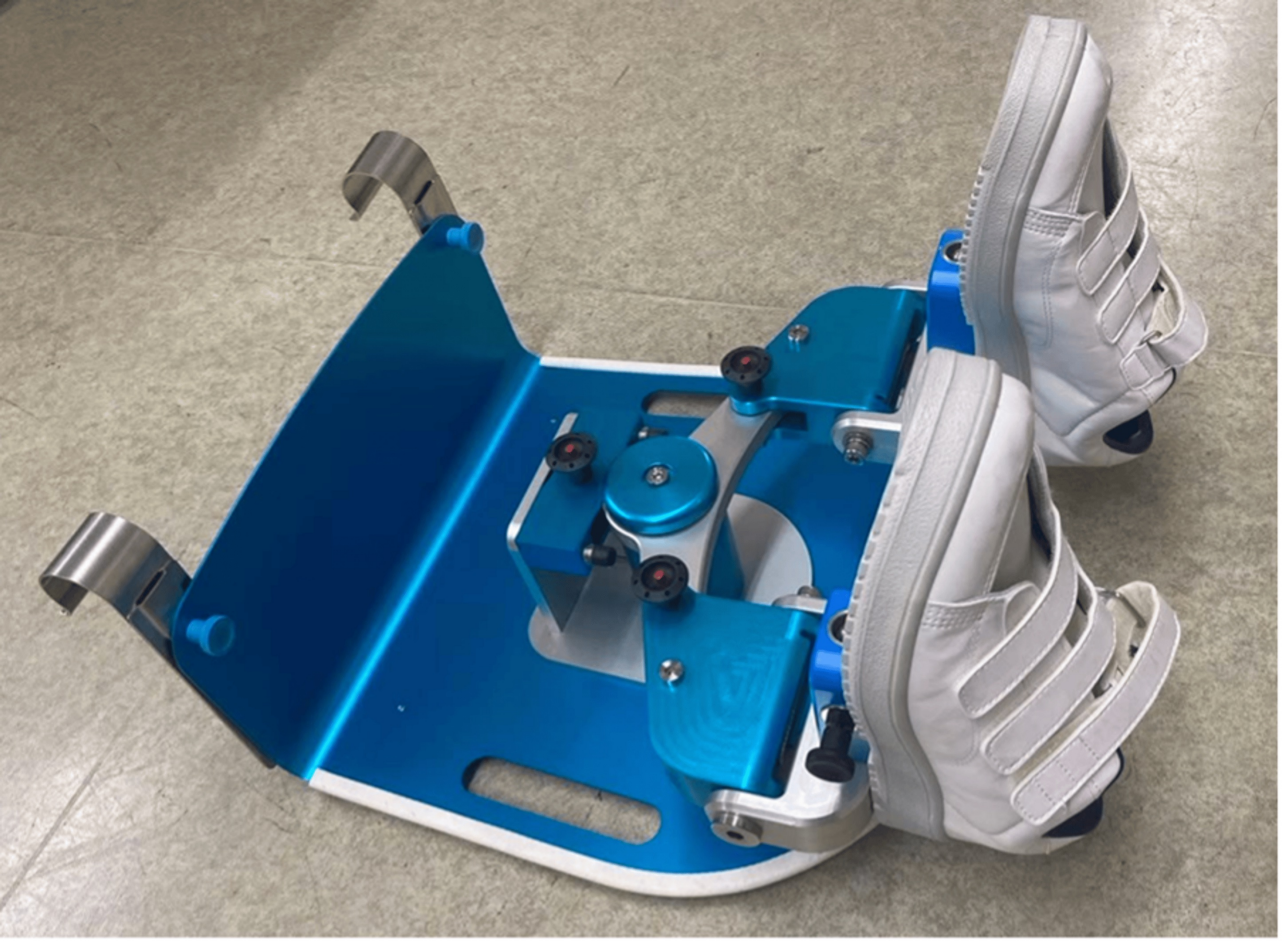
Leg exercise apparatus (LEX)

After receiving instructions on the LEX (Figure 1) and ergometer exercises, patients were evaluated for paralysis, muscle strength, sensation, range of motion (ROM), spasticity, and vital signs. It was also confirmed that the modified Borg scale (mBS) scores for respiration and lower extremity fatigue were 0 ("None") [13]. They then performed repetitive leg exercises using the LEX for five minutes while remaining in the supine position, followed by a 10-minute rest period. After confirming that the mBS scores for respiration and lower extremity fatigue had returned to 0, patients performed leg exercises for five minutes using an ergometer (Terasu Erugo, SDG Co., Ltd., Japan). The load of the variable-load ergometer was set to 20 W, as described in a previous study [12]. The order of the two exercises was fixed, with LEX performed first, as the study aimed to evaluate changes in spasticity and ROM associated with LEX exercise. Consequently, randomization and blinding were not performed.

The joint angles of the lower extremities during each exercise were not strictly controlled, allowing participants to perform the exercises comfortably. During each exercise, patients were required to perform repetitive motions at a rate of 30 repetitions per minute using a metronome at 60 bpm. If patients could not exercise independently, the examiner provided minimal manual assistance. After each exercise session, ROM, spasticity, vital signs, and mBS scores for respiration and lower extremity fatigue were re-evaluated immediately, as these parameters may have been affected by the exercises. All evaluations were conducted by the same examiner.

Upon completing all exercise sessions, patients were asked to complete a questionnaire regarding each exercise device and any adverse events. The questionnaire consisted of three questions per device: ease of use, continuability, and satisfaction. The questions were as follows: "Was the exercise easy?", "Will you be able to continue three to four sets of five minutes of exercise while in the hospital?" and "Are you satisfied with the exercise?" Patients responded using a five-point scale, ranging from one (not at all) to five (very much so). Additionally, they were asked about any new pain that developed after starting the exercise, its severity, assessed using a 0-10 Numerical Rating Scale (NRS), and whether any adverse events occurred. These two parameters were reassessed one hour after the initiation of LEX.

Outcomes

The primary outcome of this study was the occurrence of adverse events within one hour after the initiation of LEX. The secondary outcomes included paralysis of the legs, muscle strength, sensation, ROM, spasticity, vital signs, mBS scores for respiration and lower extremity fatigue, the number of exercise repetitions performed in 5 minutes, the need for assistance, and questionnaire results.

Paralysis of the lower limbs in patients with stroke was assessed using the motor items of the Stroke Impairment Assessment Set (SIAS) (SIAS-M) and the Brunnstrom Recovery Stage (BRS) [14,15]. In patients with SCI, paralysis was evaluated according to the International Standards for Neurological Classification of SCI (ISNCSCI), with measurements of the neurological level of injury (NLI) and the American Spinal Injury Association Impairment Scale (AIS) scores [16].

Muscle strength was measured using Manual Muscle Testing (MMT) for hip flexion, hip extension, knee flexion, knee extension, ankle dorsiflexion, and ankle plantar flexion. However, ankle plantar flexion was not assessed using Daniels and Worthingham’s standard method, which requires patients to stand on one leg [17]. Instead, it was measured by applying manual resistance, as with the other joints, to ensure patient safety according to the ISNCSCI evaluation.

Sensation was evaluated using the SIAS subtests for tactile and positional senses, including in patients with SCI.

ROM was measured for ankle dorsiflexion with the knee in flexion (DKF) and with the knee in extension (DKE). Spasticity was assessed using the modified Ashworth Scale (MAS) for knee flexion, knee extension, and ankle dorsiflexion [18]. Vital signs, including blood pressure (BP), pulse rate (PR), and transcutaneous oxygen saturation (SpO2), were also measured.

**Figure 2:**
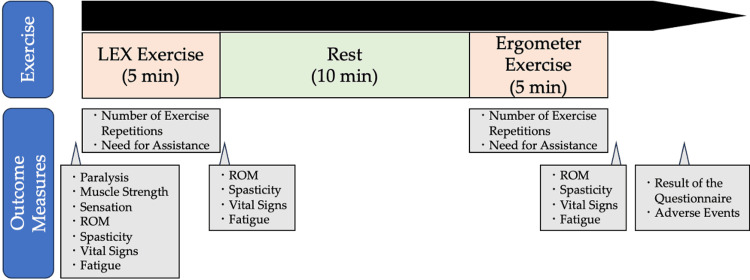
A chematic diagram of the exercises and outcome measures LEX: leg exercise apparatus; ROM: range of motion

The target number of exercise repetitions was set at 150 over five minutes; however, the actual number performed was recorded. One cycle of repetitive exercise was defined as the knee moving from maximum extension to flexion and back to maximum extension. For convenience, the number of exercise repetitions was considered "appropriate" if it fell within the range of 120 to 180. The measurement points and exercise flow are shown in Figure 2.

Additionally, patient characteristics were reviewed based on medical records, including age, sex, primary disease, disability, and locomotion items assessed using the Functional Independence Measure (FIM) [19].

## Results

Nine patients (six men and three women), aged between 41 and 74 years, were included in this study. The primary diseases included stroke in six patients-comprising subarachnoid hemorrhage, cerebral hemorrhage, and cerebellar hemorrhage, SCI in three patients, including traumatic SCI and spinal cord infarction. Among the patients with stroke, Participant 2 had the lowest SIAS-M scores, which were one, three, and two for the hip, knee, and ankle, respectively. All patients with SCI had an AIS classification of "D," indicating incomplete paralysis. Participant 9, who had SCI, exhibited the most severe sensory impairment, with bilateral tactile loss and positional sensory loss in the left limb. Regarding ROM, Participant 6, who had a stroke, had the most severe limitation on the paralyzed side, with −5° for DKE and 5° for DKF. The patient characteristics are detailed in Tables 1-3.

**Table 1:** Participants enrolled in this study M: male; F: female; SCI: spinal cord injury; FIM: The Functional Independence Measure [19]

Participant	Sex	Age	Category	Primary Disease	Disability	FIM		
						Walk	Wheelchair	Stairs
1	M	41	Stroke	Subarachnoid Hemorrhage	Left Lower Limb Paralysis	7	1	5
2	F	74	Stroke	Left Putaminal hemorrhage	Right Hemiplegia	1	6	2
3	M	47	Stroke	Right Putaminal hemorrhage	Left Hemiplegia	7	1	7
4	M	61	Stroke	Right Cerebellar hemorrhage	Right Ataxia	6	6	4
5	M	68	Stroke	Left Thalamic hemorrhage	Right Hemiplegia	5	6	5
6	F	72	Stroke	Left Thalamic hemorrhage	Right Hemiplegia	6	6	5
7	F	57	SCI	Spinal Cord Infarction	Paraplegia	1	6	1
8	M	52	SCI	Traumatic SCI	Tetraplegia	1	6	1
9	M	64	SCI	Traumatic SCI	Tetraplegia	6	6	5

**Table 2:** Characteristics of disabilities in participants with stroke The items with two numerical values were right/left. SIAS-M: motor items in the Stroke Impairment Assessment Set [14]; BRS: Brunnstrom Recovery Stage [15]; MMT: Manual Muscle Testing [17]; Flex: flexion; Ext: extension; Dorsi: dorsiflexion; Plantar: plantar flexion; ROM: range of motion; DKE: ankle joint dorsiflexion range of motion in the knee extension position; DKF: ankle joint dorsiflexion range of motion in knee flexion

Participant	SIAS-M			BRS	MMT						Sensation		ROM	
	Hip	Knee	Ankle		Hip Flex	Hip Ext	Knee Flex	Knee Ext	Dorsi	Plantar	Tactile	Positional	DKE	DKF
1	5	5	4	Ⅴ	4/4	4/4	4/4	4/4	4/4	4/4	3/3	3/3	10/5	20/15
2	1	3	2	Ⅳ	1/4	3/4	3/4	3/4	2/5	0/5	2/3	3/3	5/5	15/15
3	5	5	5	Ⅵ	5/5	5/5	5/5	5/5	5/5	5/5	3/3	3/3	5/5	20/20
4	5	5	4	Ⅵ	4/4	4/4	5/5	5/5	4/4	4/4	3/3	3/3	5/5	20/20
5	4	5	1	Ⅳ	3/5	3/4	4/5	5/5	1/5	2/5	3/3	3/3	10/10	20/20
6	4	4	3	Ⅳ	4/5	4/5	4/5	4/5	3/4	4/4	3/3	3/3	-5/10	5/20

**Table 3:** Characteristics of disabilities in participants with spinal cord injury The items with two numerical values were right/left. NLI: neurological level of injury; AIS: American Spinal Injury Association Impairment Scale [16]; MMT: Manual Muscle Testing [17]; Flex: flexion; Ext: extension; Dorsi: dorsiflexion; Plantar: plantar flexion; ROM: range of motion; DKE: ankle joint dorsiflexion range of motion in the knee extension position; DKF: ankle joint dorsiflexion range of motion in knee flexion

Participant	NLI		AIS		MMT						Sensation		ROM	
					Hip Flex	Hip Ext	Knee Flex	Knee Ext	Dorsi	Plantar	Tactile	Positional	DKE	DKF
7	Th 8		D		2/5	3/4	2/4	4/5	3/5	5/5	2/2	1/3	5/5	15/15
8	C 7		D		1/2	2/4	2/3	2/3	0/4	3/5	1/1	3/3	5/10	15/20
9	C 4		D		4/3	4/4	5/4	5/5	5/4	5/5	0/0	2/0	10/5	20/10

Adverse events, the primary outcome, were observed in two of the nine participants (Participants 7 and 8), both of whom had SCI. The adverse events involved pain, with NRS scores of two and nine. Participant 7 reported that during the LEX exercise, her right lower extremity, which had reduced muscle strength, tended to remain in ankle plantar flexion. She experienced pain when the heel of her shoe contacted the Achilles tendon area. In contrast, Participant 8 reported pain associated with preexisting spasticity in the right lower extremity, which worsened with exercise. However, the pain and spasticity resolved within one hour after exercise, and no further adverse events occurred. The remaining participants' MAS scores remained unchanged before and after each exercise (Table 4).

**Table 4:** The modified Ashworth Scale scores after each exercise Values indicate modified Ashworth Scale (MAS) scores obtained before and after each exercise [18]. Each item represents right/left. Items that did not change after the exercise were omitted. LEX: leg exercise apparatus

Participant	Knee Flexion			Knee Extension			Dorsiflexion		
	Pre-exercise	LEX	Ergometer	Before	LEX	Ergometer	Before	LEX	Ergometer
1	0/0	-	-	0/0	-	-	0/0	-	-
2	0/0	-	-	0/0	-	-	0/0	-	-
3	0/0	-	-	0/0	-	-	0/0	-	-
4	0/0	-	-	0/0	-	-	0/1	-	-
5	0/0	-	-	0/0	-	-	0/0	-	-
6	0/0	-	-	1/1	-	-	1+/0	-	-
7	1/0	-	-	1/0	-	-	1/0	-	-
8	0/0	1+/0	3/1	1+/1	1+/1	2/1+	2/1+	-	-
9	1/1	-	-	1/1	-	-	1+/1+	-	-

Two participants (Participants 2 and 7) required assistance during the LEX exercise. In Participant 2, the right hip joint tended to open and drain under gravity, and the right foot tended to fall out of the shoe, necessitating assistance to prevent these issues. As a result, Participant 2 performed the LEX intermittently. Similarly, Participant 7 required assistance to prevent the right hip joint from excessive abduction and the right ankle joint from excessive inversion. For the ergometer exercise, three participants (Participants 2, 7, and 8) required assistance. Participant 2 needed support to prevent the right hip joint from excessive abduction. Participant 7 required assistance to prevent reverse pedaling when transitioning from maximum knee flexion to extension. Additionally, significant shifting of the ergometer occurred due to lower limb movement, requiring assistance for fixation. Participant 8 also required assistance in a similar manner to prevent reverse pedaling during both maximum knee extension and flexion. Regarding the number of exercise repetitions, eight participants were classified as "Appropriate" for the LEX exercise, whereas only four were classified as "Appropriate" for the ergometer exercise. Participant 7 required substantial assistance, and at her request, the ergometer exercise was discontinued after three minutes and 10 seconds. The results of these exercises are presented in Table 5.

**Table 5:** Results of each exercise The evaluation was "Appropriate" if the number of exercise repetitions was 120–180, "Below" if the number of exercise repetitions was less than 120, "Above" if the number of exercise repetitions was more than 180, and "Unable" if the exercise could not be completed. If the patient required assistance during the exercise, "+" is noted. LEX: leg exercise apparatus

Participant	LEX			Ergometer		
	Number of Exercise Repetitions	Evaluation	Assistance	Number of Exercise Repetitions	Evaluation	Assistance
1	150	Appropriate	-	151	Appropriate	-
2	91	Below	+	96	Below	+
3	150	Appropriate	-	151	Appropriate	-
4	137	Appropriate	-	195	Above	-
5	154	Appropriate	-	131	Appropriate	-
6	140	Appropriate	-	110	Below	-
7	148	Appropriate	+	71 (Stopped Early)	Unable	+
8	141	Appropriate	-	78	Below	+
9	148	Appropriate	-	150	Appropriate	-

Regarding mBS scores for respiratory fatigue, three out of nine participants (Participants 2, 3, and 7) reported that the ergometer exercise resulted in a score one level higher than the LEX exercise. For lower extremity fatigue, five participants (Participants 2, 3, 6, 7, and 8) reported that the ergometer exercise resulted in a score one level higher than the LEX exercise. The results are shown in Tables 6, 7.

**Table 6:** The mBS scores for respiration fatigue LEX: leg exercise apparatus; mBS: modified Borg scale [13]

Participant	LEX	Ergometer
1	0	0
2	1	2
3	0.5	1
4	1	1
5	0	0
6	0	0
7	0.5	1
8	4	4
9	3	3

**Table 7:** The mBS scores for lower extremity fatigue LEX: leg exercise apparatus; mBS: modified Borg scale [13]

Participant	LEX	Ergometer
1	0	0
2	0.5	1
3	0.5	1
4	1	1
5	0	0
6	0	0.5
7	1	2
8	6	7
9	4	4

**Figure 3:**
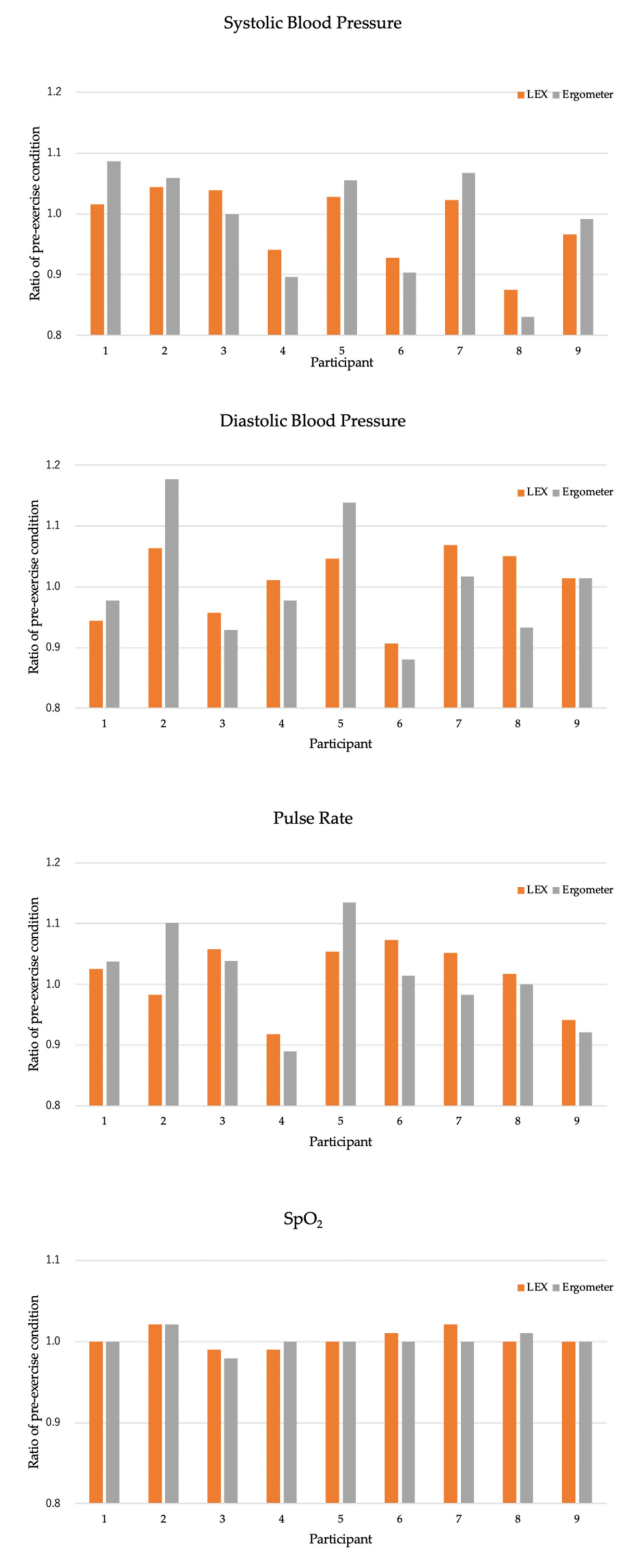
Changes in vital signs The vertical axis represents the ratio of the values at rest. LEX: leg exercise apparatus; SpO_2_: saturation of percutaneous oxygen

ROM and vital signs did not change significantly after each exercise (Figure 3).

**Figure 4:**
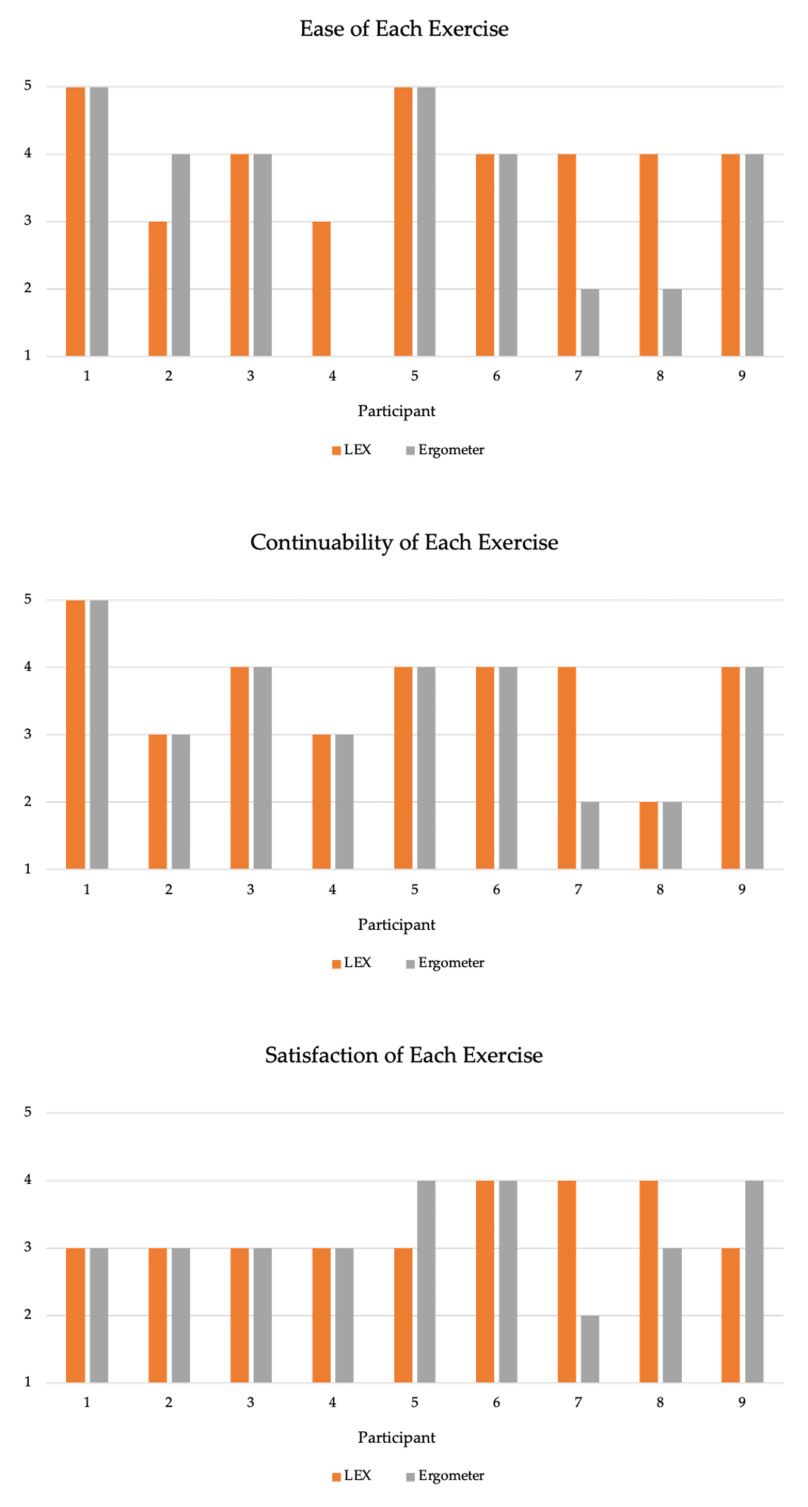
Results of the questionnaire The vertical axis represents the score of the responses to the questions, expressed on a five-point scale from one (not at all) to five (very much so). LEX: leg exercise apparatus

Regarding the questionnaire results, one participant with stroke (Participant 2) found the ergometer exercise easier, whereas three participants (Participants 4, 7, and 8) found the LEX exercise easier. According to most participants, there was no difference in the ability to continue using the two devices. However, participant 7 with SCI, who could not complete the ergometer exercise, reported that the LEX exercise was more sustainable. As for satisfaction, only participant 7 reported more satisfaction with the LEX exercise. The results are shown in Figure 4.

## Discussion

In this study, the safety of LEX was evaluated in patients with stroke or SCI, both of which affect the central nervous system. Adverse events, the primary outcome, were observed in two patients; however, these events were not serious and resolved within one hour. Since participants performed both the LEX and ergometer exercises, it was impossible to determine whether these adverse events were caused by one specific exercise. However, Participant 7 reported discomfort caused by shoe contact with the heel during the LEX exercise, suggesting that this issue was directly related to LEX. Given that LEX is intended for patients with limited voluntary control of the ankle joint, incorporating a mechanism to maintain proper foot positioning may improve its usability and comfort.

For Participant 8, knee spasticity temporarily worsened and was accompanied by severe pain. This participant had relatively greater spasticity before exercise than the other participants. The severity of pre-exercise spasticity may contribute to its exacerbation post-exercise, indicating that greater caution is needed when using LEX for such patients. However, previous research on the effects of exercise on spasticity in patients with stroke suggests that continued exercise does not necessarily worsen spasticity but may instead maintain or reduce it [20]. Therefore, the impact of LEX on spasticity should be further investigated through long-term interventional trials.

Regarding vital signs, no significant changes were observed after LEX, consistent with previous studies [6,9]. In patients with stroke, circulatory dynamics should be carefully monitored. Additionally, patients with SCI may experience hypotension due to sympathetic nerve dysfunction or marked increases in blood pressure triggered by stimulation of a paralyzed area, necessitating similar vigilance in monitoring circulatory dynamics [21]. Previous studies have shown that LEX exercise does not induce excessive circulatory changes in healthy individuals [12]. The findings of the present study suggest that LEX can also be safely used in patients with stroke or SCI.

Seven participants, including those with stroke and SCI, were able to perform an "Appropriate" number of exercise repetitions with LEX. This group included Participant 6, who had the most severe ROM limitation, and Participant 9, who had the most severe sensory disorder. This indicates that sensory disorders and mild ROM limitations alone do not affect adaptation to LEX. Although Participant 7 with SCI required assistance to perform LEX, Participant 8 with SCI, who had weaker or similar muscle strength, could perform the exercise without assistance. Since both participants who required assistance needed support to prevent the hip joint from excessive abduction, muscle strength not measured in this study, such as internal rotation and adduction muscles, may play a crucial role in determining adaptability to LEX.

During the ergometer exercise, two participants required assistance to prevent reverse rotation. Both participants had SCI, which suggests that bilateral lower extremity impairment prevented them from compensating with the nonparalyzed side, as in patients with stroke. However, unlike the vertical rotational motion of the ergometer, LEX exercises involve horizontal back-and-forth foot movements. As a result, structural issues related to reverse rotation do not arise, making LEX potentially more suitable for patients with paralysis in both lower extremities, such as those with SCI. This may explain why Participants 7 and 8 with SCI found LEX easier to perform.

Regarding mBS scores, fatigue in the respiratory system and lower extremities was less pronounced during LEX compared to the ergometer exercise. Interestingly, a previous study on healthy participants found that LEX led to greater lower extremity fatigue, which contrasts with the present study's findings [12]. As mentioned earlier, this discrepancy may stem from the difficulty patients with motor impairments experience controlling rotational movements on an ergometer. LEX may provide a more suitable option for sustained exercise in these patients.

The ease of performing exercises with the ergometer was notably low, and the number of exercise repetitions was excessive for Participant 4, who had ataxia, making it difficult to maintain a consistent pace. Participants who failed to achieve the appropriate number of exercise repetitions were more frequently observed with the ergometer than with LEX. This finding suggests that LEX may be easier to perform at a constant pace for patients with motor disorders and may offer the advantage of stabilizing the load when used in exercise therapy.

This study had several limitations. First, the carryover or fatigue effects were not well controlled. The study aimed to evaluate changes in spasticity and ROM associated with LEX exercise by having participants perform LEX first. However, this sequencing may have influenced the outcomes observed during the subsequent ergometer exercise. Second, participant characteristics were heterogeneous. As a pilot study, no restrictions were placed on disorder status. While both stroke and SCI are central nervous system diseases, they result in distinct disabilities; stroke typically causes hemiplegia, whereas SCI is more likely to lead to paraplegia or tetraplegia. Moreover, even among individuals with the same condition, the degree of disability varies. To statistically verify the effects of LEX in future research, a randomized controlled trial should be conducted with participants from the same disease group, ensuring greater uniformity in their characteristics. Third, this study did not include participants with relatively severe disabilities, such as patients with stroke with a BRS of III or lower, or patients with SCI with an AIS classification of A to C. Further research is needed to assess the feasibility of LEX in patients with more severe disabilities.

Despite these limitations, this pilot study provides preliminary evidence supporting the safety and feasibility of LEX in patients with motor impairments. Future studies with more homogeneous populations stratified by diagnosis and impairment severity and rigorous designs are recommended to validate these findings and explore the long-term rehabilitative potential of LEX.

## Conclusions

This study represents the first trial of the LEX exercise in patients with motor disabilities due to stroke or SCI. No adverse events were observed except for transient pain symptoms, indicating that LEX exercises can be performed safely. However, the possibility of temporary worsening of spasticity should be considered. Additionally, LEX exercise may not be suitable for patients with weak hip internal rotation and adduction muscles who are unable to maintain a midline position when flexing their lower extremities in the supine position. With slight modifications to improve foot stability and prevent excessive hip abduction, LEX may serve as a practical and effective exercise modality for lower limb rehabilitation in patients with motor impairments.
